# Effect of Different Surface Treatments on Repair Bond Strength of CAD/CAM Resin-Matrix Ceramics

**DOI:** 10.3390/ma15186314

**Published:** 2022-09-12

**Authors:** Semih Arkoy, Mutahhar Ulusoy

**Affiliations:** Department of Prosthodontics, Faculty of Dentistry, Near East University, Near East Boulevard, Mersin 10, Nicosia 99138, Turkey

**Keywords:** airborne particle abrasion, Er,Cr:YSGG laser, micro-tensile bond strength, nanohybrid composite, resin-matrix ceramic

## Abstract

The purpose of this study was to investigate the influence of different surface treatment methods on the micro-tensile bond strength (μTBS) of resin-matrix ceramic (RMC) blocks repaired with resin composite. Three different prefabricated RMC blocks including Lava Ultimate (LU), Grandio Blocs (GB), and Shofu Block HC (HC) were thermo-cycled and divided into five surface treatment groups: Control (C), bur grinding (G), airborne particle abrasion (APA), Er,Cr:YSGG laser irritation (LI), and APA combined with LI (APA+LI). After surface treatments, topographic alterations were examined by scanning electron microscopy. Then, Universal Adhesive (Single Bond Universal) was applied and repair was simulated with nanohybrid composite (Grandio SO). Bonded specimens were cut into 1 mm^2^ sized beams (*n* = 16) and a μTBS test was conducted by using a universal test machine. Fracture types were evaluated by using a stereomicroscope. The bond- strength data was evaluated by two-way ANOVA and Tukey post-hoc test (α = 0.05). The μTBS values were significantly affected by the surface treatment variable and the interaction terms of the variables (*p* ≤ 0.001). However, no significant effect of RMC type was detected (*p* > 0.05). Among all materials, GB^APA+LI^ indicated the highest µTBS value. Except for the GB^C^, all surface treatments showed clinically acceptable bond-strength values. However, the surface treatments applied to GB and LU before the repair processes increased the repair bond-strength values while causing a negative effect for HC. In addition, LI and APA+LI can be applied as an alternative route compared to other procedures recommended by the manufacturer for surface preparation in intraoral RMC repair.

## 1. Introduction

Increasing aesthetic demands of patients has led to the development of all-ceramic indirect restorations. Although these restorations provide a successful service in the replication of the optical properties of a natural tooth, they present low tensile and fracture strength [1].

The use of computer-aided design/computer-aided manufacturing (CAD/CAM) restorations has increased as they can be manufactured chairside in a single session [2]. Moreover, CAD/CAM restorations exhibit better mechanical properties compared to those produced by the conventional route [3]. In contemporary dentistry, manifold CAD/CAM blocks depicting different characteristics are available on the dental market. Of these, the attention surrounding resin-matrix ceramics (RMCs) have latterly increased. RMCs allow the integration of the advantageous features of ceramics and composite resins [4]. RMCs are easier to mill due to the soft matrix structure. They can be repaired intraorally and can provide enhanced marginal fit, superb mechanical and physical properties, and better fracture resistance compared to direct composite restorations [2]. Moreover, RMCs successfully emulate the physical properties of the natural tooth [5]. Lava Ultimate (LU), Shofu Block HC (HC), and Grandio Blocs (GB) are some of the currently available RMC blocks. LU consists of zirconium and nano silica particles (80%) embedded in a polymeric matrix (20%). HC consists of more than 61% by mass of inorganic filler particles, silica powder, zirconium silicate, and micro-clustered silica. GB has a high filler content of 86% among other RMCs [6,7,8,9,10].

Intraoral repair of defective restorations with composite resin is commonly encountered in clinical practice as this route offers low cost, is more conservative, can be done in a single session, results in less tooth tissue loss, and causes less pulpal trauma, compared to the removal and renewal of the indirect restoration [11]. Although stiff restorative materials exhibit enduring mechanical properties, they are more susceptible to fracture under masticatory forces due to the decreased flexural strength [12]. The different chemical composition of CAD/CAM materials used in restorative procedures may also influence their repair potential [13]. Unlike conventional ceramic restorations, it is very difficult to repair CAD/CAM restorations because of their industrial route of polymerization under high pressure and temperature. This route presents a high degree of conversion (up to 95%) [14], thereby decreasing the number of carbon–carbon double bonds and making intraoral repair more difficult. With this regard, before repair, these restorations necessitate the implementation of dedicated surface treatments [15]. Since the characteristics of RMCs vary, there is still no consensus not only on the repair method but also on the ideal surface treatment [7,11,16,17].

As no standard adhesive resin material for the repairment of the restorations is available, this should be performed by silane coating agents combined with surface treatments [18,19]. The micromechanical interlocking and chemical adhesion between the repair composite and the fractured restoration is a crucial factor in the repair procedure [20]. In previous studies, various surface treatments such as grinding with diamond burs or silicone carbide discs, tribo-chemical coating, aluminum oxide airborne particle abrasion, acid etching, silane coupling agent, and adhesive systems have been shown to increase the repair bond strength of the resin composite to the RMCs [21,22,23]. Alternatively, the use of a universal adhesive on the materials to be repaired allows the achievement of chemical bonding through the ionic interaction between the composite resin particles and the acidic group of the functional monomers of the material [11]. In addition to conventional functional monomers, these adhesives contain phosphate monomer (methacrydiyloxydyl dihydrogen phosphate [MDP]), and silane [24]. Silane is used as a bonding agent because it interacts with the crystalline portion of the ceramic and the organic portion of the composite resin [25]. Laser irradiation is also recommended as an alternative surface-treating strategy for the restorations made of composites, ceramics or RMCs [13]. Supportively, in order to obtain micromechanical retention by increasing the surface roughness of the restorative material (neodymium garnet yttrium aluminum (Nd:YAG), erbium-doped yttrium aluminum garnet (Er:YAG), and erbium, chromium: yttrium-scandium-gallium-garnet (Er,Cr:YSGG), lasers are preferred by many researchers [26,27]. The applied laser beam released from the laser system itself is absorbed by the water, and as a result of micro explosions, particles are removed from the material surface, specifically from the inorganic portion, and microporosities are generated [28].

Although the intraoral repairment of all-ceramic restorations has been extensively investigated in the literature [12,23,26,27]; the scientific data regarding the surface treatments and intraoral repair protocol of RMCs are sparse. Therefore, it was purposed to scrutinize the influence of varied surface treating protocols on the micro-tensile bond strength (μTBS) between the thermally-aged RMCs and the repair composite. The first hypothesis was that RMC type would influence the μTBS values, and the second hypothesis was that surface treatments would become influential on the μTBS of the experimental groups.

## 2. Materials and Methods

All materials used in this study are given in Table 1.

**Figure 1:**
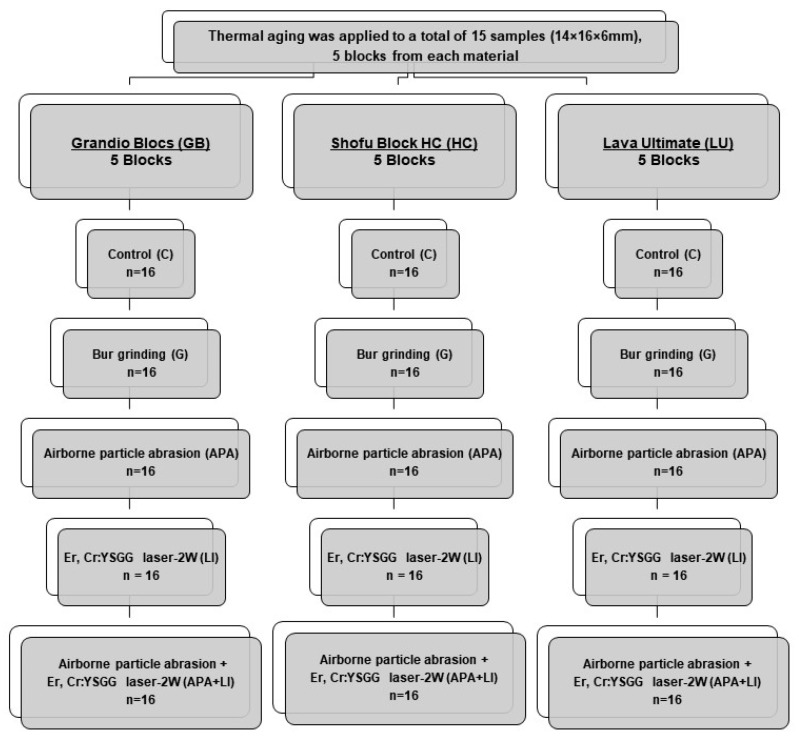
Schematic diagram of five surface treatments applied to each evaluated RMCs.

Three different RMCs (GB [Voco], HC [Shofu Dental], and LU [3M ESPE]) were sectioned by using a water-cooled high speed precision cutting machine (Microcut 201, Metkon, Bursa, Turkey) to obtain 5 14 × 16 × 6 mm^3^ blocks. These blocks were then subjected to thermal aging (Thermocycler, THE-1100, SD Mechatronik, Feldkrichen-Westerham, Germany) to simulate a 1-year usage in the oral environment (×10,000, dwell time of 20 s, between 5 °C and 55 °C) [29,30,31]. After the aging process, the intaglio surface of each specimen was abraded with a 600-grit silicone carbide abrasive paper to achieve a standardized substrate surface (20 s, under low pressure and cold-water irrigation) [32], followed by ultrasonic cleaning (ISOLAB, Laborgerate GmbH, Eschau, Germany) for 10 min in distilled water. The thermally-aged specimens were kept on hold in distilled water at 37 °C until the surface treatments were applied (Shaking Water Bath, type 3047, Kötterman, Hänigsen, Germany). Subsequently, each RMC material was further divided into 5 subgroups in accordance with the applied surface treatments: Control (C), bur grinding (G), aluminum oxide (Al_2_O_3_) airborne particle abrasion (APA), Er,Cr:YSGG-2W laser irradiation (LI), and airborne particle abrasion coupled with Er,Cr:YSGG-2W laser irradiation (APA+LI). All surface treatments were applied by a single calibrated clinician (Figure 1).

For the C group, no surface treatment was conducted. For the G group, the bonding surface was roughened under low hand pressure with a green belt diamond bur for 15 s with 15 perpendicular and 15 horizontal movements [13]. In the APA group, 50 µm Al_2_O_3_ particles (Korox, Bego, Bremen, Germany) were perpendicularly thrown towards the bonding surface of the specimen from a distance of 10 mm for 20 s at 3 bar pressure (Rotaks-Dent, Istanbul, Turkey). In the LI group, Er,Cr:YSGG laser (Waterlase MD, Biolase, Irvine, CA, USA) irradiation was performed in a noncontact hard tissue mode with an MG6 sapphire tip at an energy level of 2W, 140 ms pulse duration and a repetition rate of 10 Hz with 55% water and 65% air for 20 sec. In the APA+LI group, the above-described surface-treating strategies were applied, respectively.

One specimen from each group was dried, coated with palladium (SC7620, Emitech Mini Sputter Coater, Quorum Technologies, East Sussex, UK) and examined under scanning electron microscope (SEM)(JSM-6610LV, JEOL, Tokyo, Japan) at ×1000 magnification to understand the topographic alterations caused by the surface treatments. These specimens were only used for SEM analysis and not used in any other part of the study.

Subsequent to surface treatments, ultrasonic cleaning of all specimens was done with distilled water for 10 min and then specimens were placed on a base of acrylic resin (Self cure, Imicryl, Konya, Turkey) in order to make it possible to precisely adapt to the precision cutting device. Universal adhesive (Single Bond Universal, 3M ESPE, St. Paul, MN, USA) was applied for 20 s on the upper roughened surface of each specimen on the acrylic resin, and, after air-drying for 5 s, it was light-polymerized (LED-B, Woodpecker, 1000–1700 mw/cm^2^) for 10 s. The specimens were placed into a silicone matrix made of poly vinyl siloxane (Elite HD+ Putty Soft, Zhermack, Polesine, Italy) and prepared in dimensions of 14 × 16 × 12 mm^3^. Grandio SO (VOCO) repair composite was incrementally applied as of 2-mm-thickness. The same silicone matrix was used to achieve standardization for each repaired specimen. Each layer of repair composite was polymerized from the top with a LED light device for 20 s. Subsequent to polymerization, the ceramic-composite resin blocks were removed from the silicone matrix and light-cured for an additional 40 s from the side surfaces to achieve complete polymerization of the adhesive surface. The specimens were then kept in distilled water at 37 °C for 24 h.

**Figure 2:**
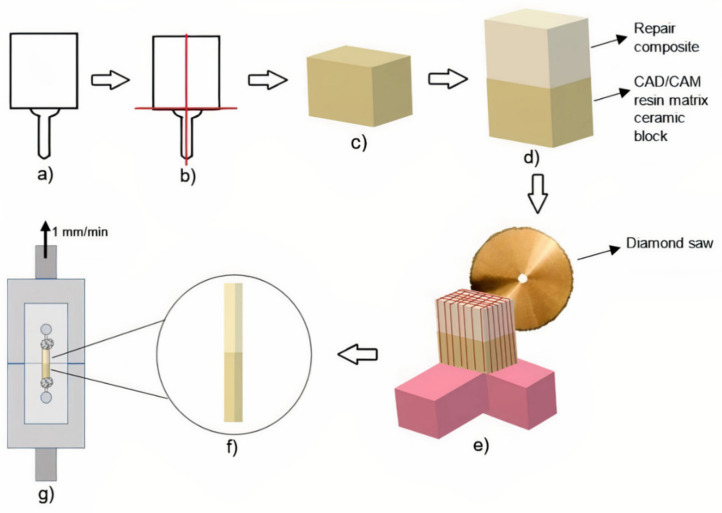
Preparation steps of RMC blocks for micro-tensile bond strength test: (**a**) RMC block; (**b**) Blocks sectioned into two parts by cutting horizontally and vertically with precision cutting machine; (**c**) Resulting block with dimensions of 14 × 16 × 6 mm^3^; (**d**) Application of 6 mm repair composite onto RMC block; (**e**) Horizontal and vertical cuts made with 1 mm intervals with precision cutting machine; (**f**) Non-trimmed bar specimens reduced to 1 × 1 × 12 mm^3^ dimension; (**g**) Micro-tensile test performed by sticking the specimens to the Geraldeli’s Jig from both ends with cyanoacrylate resin.

The repair-composite-applied specimens were fixed to the precision cutting device by the acrylic resin holder edges. With this device, horizontal and vertical cuts were made with 1 mm intervals. Only central beams were used in the post-cutting micro-tensile bond strength test, peripheral beams were excluded. Thus, the results were not influenced by the excessive or insufficient amount of resin composite at the margins. Therefore, 1.0 ± 0.1 mm^2^ non-trimmed bar-shaped specimens were obtained. Each non-trimmed bar-shaped specimen was sectioned into slices (n = 16) depicting the dimension of 1 × 1 × 12 mm^3^, scrutinized with the help of a digital caliper, and the measured dimensions were recorded before the μTBS test. The specimens were adhered to the test apparatus from both ends with two drops of cyanoacrylate resin (EUROFIX, Istanbul, Turkey) in order to perform the μTBS test, as described by Raposo et al [33]. The μTBS test was conducted with the aid of Geraldeli’s Jig in a universal testing device (EZ 50, LLOYD Instruments, Ametek Inc., West Sussex, UK) at a speed of 1 mm/min (Figure 2).

**Figure 3:**
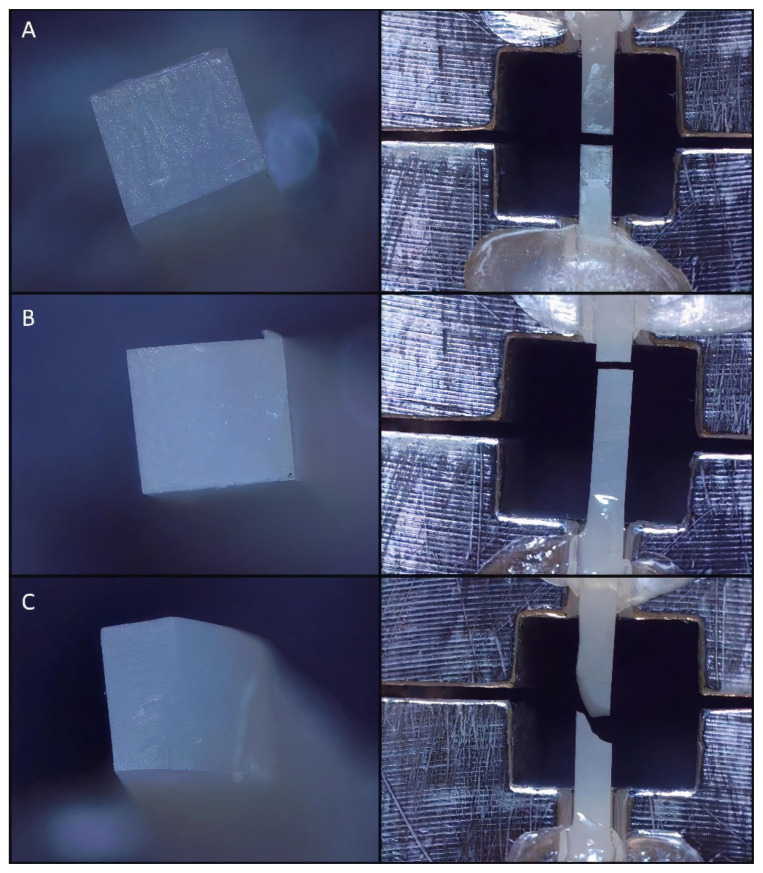
Different failure modes (×40 magnification). (**A**) adhesive, (**B**) cohesive, (**C**) mixed.

The force required to fracture the specimens was recorded in Newtons (n). A digital caliper was used to measure the cross-sectional area of the adhesive site. The μTBS (MPa) of each specimen was calculated by using the following function: *μTBS = failure load (N) ⁄ cross-sectional area (mm^2^) of the test beam*. Afterwards, a stereomicroscope (Olympus SZ61TR, Olympus Corporation, Shinjuku, Tokyo, Japan) at ×40 magnification was used to examine the fractured beams in order to determine the failure modes (either adhesive, cohesive, or mixed) (Figure 3). Failure modes were categorized as follows: adhesive type failure is the type of failure seen at the resin composite and RMC interface; direct resin composite cohesive failure is the failure mode whose boundaries remain within the composite resin; RMC cohesive failure is failure seen on RMC materials; mixed failure is a failure condition in which adhesive and cohesive failure of the resin composite or RMCs occur together [33].

For statistical analyses, a mathematical software (IBM SPSS Statistics, v23, IBM Corp., Chicago, IL, USA) was used. Normality of data distribution was ratified with the Shapiro–Wilk test (*p* > 0.05). Therefore, to study the influence of RMC type and surface treatment variables on the μTBS data, 2-way analysis of variance (2-way ANOVA) and the Tukey post hoc tests were performed. A statistical significance level was taken as α = 0.05.

## 3. Results

The 2-way ANOVA results depicted that μTBS values were significantly affected by the surface treatment variable and the interaction terms of the variables (*p* ≤ 0.001). For the main effect of surface treatment, the highest μTBS value was obtained in APA+LI (63.02 ± 13.54)^Z^, followed by LI (58.30 ± 11.58)^Y,Z^, G (57.54 ± 19.87)^Y,Z^, APA (54.08 ± 19.04)^Y^ and C (36.94 ± 23.09)^X^, respectively. However, for the main effect of RMC type, no significant effect was detected (*p* > 0.05) (Table 2). According to the material, the total mean values were 55.83 ± 7.95 for GB, 52.43 ± 15.88 for HC and 53.65 ± 14.21 for LU. The mean μTBS ± standard deviations including Tukey post hoc comparisons are given in Table 3.

Among all experimental groups, GB^APA+LI^ indicated the highest µTBS value (70.94 ± 6.03). On the other hand, GB^C^ showed the lowest µTBS value (18.01 ± 7.88). Considering the C group for all three materials, all surface treatments significantly increased the repair µTBS values in GB and LU material, while µTBS values were significantly decreased in the HC^G^ and HC^APA^ groups of HC material (*p* < 0.05). Considering the µTBS values of the C, G, and APA groups, the HC material was significantly different from other RMCs. In the HC group, HC^C^ had the highest µTBS values, while HC^G^ and HC^APA^ treatment decreased µTBS values. Except for GB^C^ and LU^C^, there was no significant difference between the surface treatments of GB and LU material (*p* > 0.05). Within LU, the µTBS value of C group was significantly lower than those of surface-treated specimens. Among the surface-treated LU specimens, the µTBS value of LU^G^ was significantly higher, while the LU^LI^ treatment showed the lowest µTBS values.

**Figure 4:**
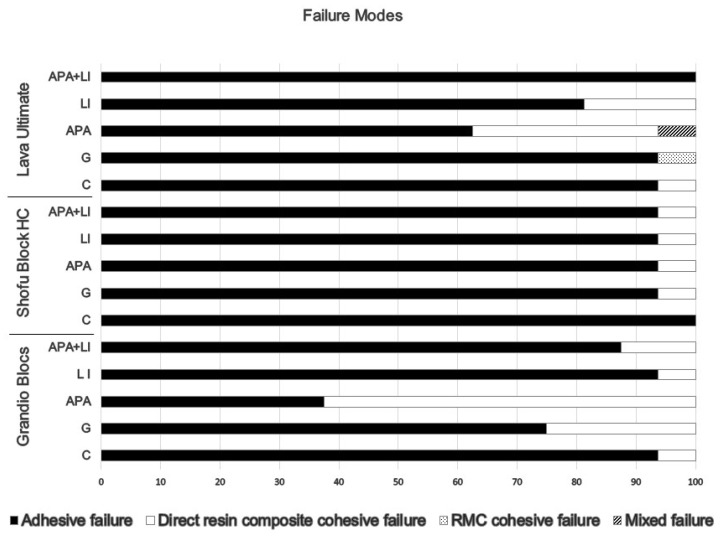
Failure mode distributions (%) of specimens subjected to micro-tensile bond strength test.

The percentages of the failure modes that occurred in the test groups are given in Figure 4.

It was seen that the dominant failure mode was adhesive in all subgroups of RMCs, except for the GB^APA^ group in which direct resin composite cohesive failure was predominantly observed (62.50%).

**Figure 5:**
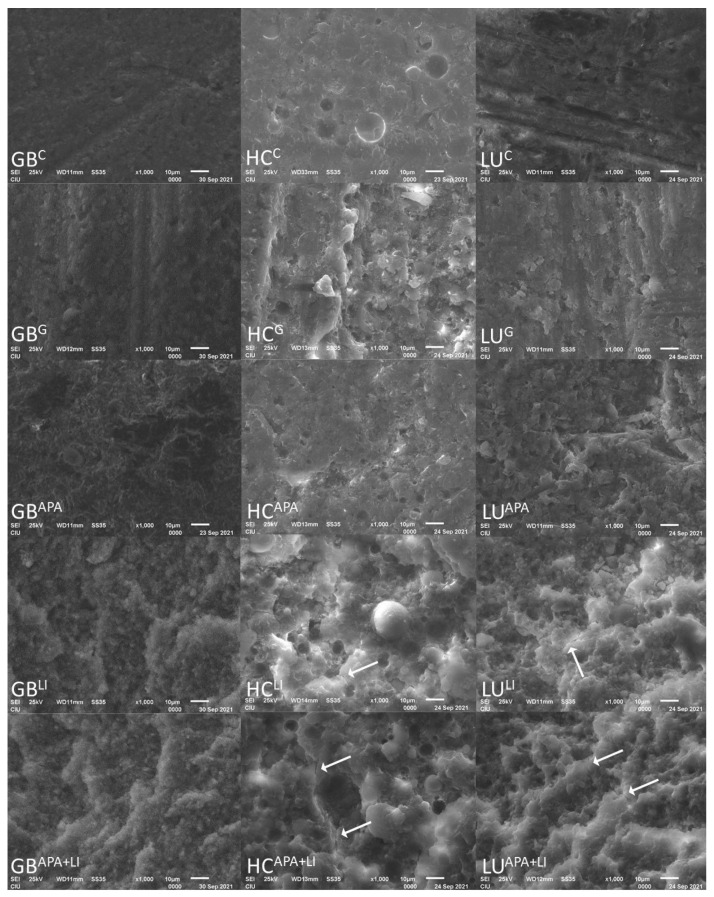
Scanning electron micrographs of specimens (×1000 magnification): GB, Grandio Blocs; HC, Shofu Block HC; LU, Lava Ultimate; C, Control; G, Bur grinding; APA, Airborne particle abrasion; LI, Er,Cr:YSGG laser irradiation; APA+LI, Airborne particle abrasion + Er,Cr:YSGG laser irradiation. White arrows indicate crack lines.

The SEM images of the RMCs conditioned with different surface treatment protocols are presented in Figure 5. In the interpretation, surface fluctuations and irregularities were observed especially after the APA, LI, and APA+LI surface treatments. In the LI and APA+LI groups, irregular particles and deep pits were also observed related to the explosions created by the laser irradiation. In addition, crack lines (white arrows) were observed especially in the LI and APA+LI groups.

## 4. Discussion

As reliable bonding is a cumber-stone in the long-term viability of the restorations, this comparative study investigated the influences of different surface treatment protocols on the surface characteristics and the repair µTBS of aged RMCs. The results of the 2-way ANOVA proved that μTBS values were significantly influenced not only by the surface treatment but also by the interaction term. However, the RMC type did not significantly alter the results. Therefore, the first hypothesis was rejected and second hypothesis was accepted.

The micro-topography of the composite surfaces that are grinded with diamond burs, penetrated with APA by using aluminum oxide abrasives, or tribo-chemical silica-coated has been comprehensively investigated with the aid of SEM in previous studies [34,35], illustrating that the mechanical surface treatments established a micro-retentive surface which makes micromechanical interlocking of the adhesive resin feasible. To the best knowledge of these authors, there is no consensus on the RMCs repair bond strength, and controversial results have been reported [7,13,18,36] For various RMCs, such as LU and HC, most manufacturers recommend the sand-blasting of the intaglio surface [37]. Supportively, APA has been shown to improve bond strength by removing the smear layer formed after milling, revealing a fresh contaminant free surface and providing enhanced micromechanical retention. However, this procedure also reveals a tendency towards 1–10 µm surface damage and micro-cracking [19,38]. Yoshihara et al. [39] showed that these cracks can be detected within the resin-matrix and at the interface between the resin-matrix and the filler particle. In this study, all surface treatments applied to GB and LU increased the μTBS values compared to the C group. However, lower μTBS values were observed in the HC^G^ and HC^APA^ groups compared to other surface treatments. These differences in μTBS values may be due to possible cracks in the matrix or low surface energy due to the smear layer.

RMC blocks show less chipping after bur grinding with respect to their higher stress absorption capacity and lower modulus of elasticity [40]. According to the study of Bayraktar et al [13], bur grinding showed the highest values for efficient repair μTBS to RMC blocks compared to hydrofluoric acid etching, Er:YAG, Er,Cr:YSGG, and Nd:YAG laser irradiations. In the study conducted by Kimyai et al [41] on laboratory composites, it was found that 2W Er,Cr:YSGG application showed more effective bond strength compared to diamond milling. Erdemir et al [23] proved that high roughness values were acquired with the diamond bur, but the implementation did not increase the bond strength. This finding has been attributed to the idea that the roughening with the bur is on a macro-scale and the geometry of the roughened surface is more important for micromechanical bonding. However, considering the SEM images in this study, acceptable bond strength values were obtained although all bur grinding groups supported roughening at macro-scale [32].

Laser irradiation provides a good service in the enhancement of bond strength [13]. However, alterations in laser model, inter-distance between the nozzle and the substrate surface, power output, or irradiation time may cause differences in surface topography [42]. Er, Cr:YSGG lasers have been commonly preferred in previous studies, as the risk of the formation of a heat-damaged layer is lessened due to its hydrokinetic structure and the fact that it does not form a smear layer [43]. In addition, Er, Cr:YSGG lasers can be contemplated as a safer alternative mechanical treatment because the effects of Al_2_O_3_ aerosols used in air-etching methods on the human body are detrimental. APA has been proven to be a successful surface treatment in previous studies, and data were sufficient [6,7,11] Thereby, APA was coupled with LI in this study to better understand their cumulative effects. Moreover, the highest µTBS values in GB were obtained in the APA+LI group. In all RMCs, the APA+LI surface treated groups showed as successful or higher µTBS values as the LI group.

Oz et al [36] evaluated the composite bond strength of surface treatments on hybrid CAD/CAM blocks and found no statistically significant difference between sandblasting, 2W Er, Cr:YSGG, and 3W Er, Cr:YSGG. Although there were similar results between LI and APA in this study, APA+ LI values were found to be significantly higher than the APA group except for the LU material. In the APA process, the surface roughness and surface area are significantly increased with 50 µm aluminum oxide particles first applied to the surface under pressure [18] Laser micro explosions on the material surface, whose surface area has already been increased by applying APA, create deeper pits in addition to micron-sized surface irregularities. This is also supported by the SEM images in our study. The laser also provides the removal of the glass phase in the material, unlike APA [44] Bayraktar et al [13] observed that the repair capacity of the material increased as the resin content increased. This may explain why the GB^APA+LI^ group, which has the highest resin content, has the highest bond strength values.

In LU, LU^G^ and LU^APA^ exhibited higher µTBS values than other surface treatments. The bonding behavior of the LU depends on several factors. First, it contains a high percentage of ZrO_2_ (zirconium dioxide). According to the Vickers Hardness Scale, Al_2_O_3_ (2000) offers superior hardness to ZrO_2_ (1200) [45]. Thus, during abrasion, Al_2_O_3_ abrasives become more effective and form an active surface, providing strong adhesion. During APA, only chemicals with a hardness less than the Al_2_O_3_ (2000) particle hardness are removed from the ceramic surface. However, in LI, the entire inorganic portion of the surface is affected [28]. This may weaken the connection between silane and ceramic. This may have caused the LU^G^ and LU^APA^ to show higher µTBS values compared to LU^LI^. Due to the lower microhardness of LU than GB, laser irritation may have caused detrimental effects on the inorganic structure of the material [46]. High nanohybrid filler content (86% by weight) of GB, may explain the higher µTBS values of the GB^LI^ and GB^APA+LI^ groups compared to other surface treatments. 

RMCs show a high degree of conversion than direct composites, but it is not feasible to achieve 100% conversion from the monomeric to the polymeric phase, and some free monomers remain embedded in the structure [47]. However, the resin ratios of the materials are 14% for GB, 20% for LU, and 39% for HC [6,7,8,10]. While all three materials contain UDMA (urethane dimethacrylate) in resin structure, HC and LU also include TEGDMA (triethyleneglycol dimethacrylate); unlike GB [9], TEGDMA increases the degree of conversion compared to UDMA [48]. Therefore, GB may contain more free monomers than other materials. The generation of covalent bonding between the material to be repaired and the repair resin can be practicable due to the presence of unpolymerized carbon–carbon double bonds [49]. 

Michelotti et al [50] shows that the silane-containing universal adhesive used with or without separate silanization has a similar effect on the composite-to-composite repair bond strength. In addition, ultra-mild adhesives such as Single Bond Universal (SBU) (pH = 2.7) are less inclined to hydrolytic degradation than more acidic systems, leading to affirmative bonding stability. SBU, preferred to be used in this study, contains 10-MDP in its formulation. 10-MDP is a sticky functional monomer that increases the potential of the surface to chemically bond to metals, zirconia and dental tissues by forming insoluble calcium salts [51]. Yoshihara et al [38] showed that filler silanization before bonding increased the mechanical properties of the composite, and they also argued that it could not be adequately silanized due to spherical fillers in HC content. In this study, the reason for the high µTBS values (64.35) of HC^C^ can be explained by the fact that the material has a resin content of 39% and sufficient chemical bonds are formed between the zirconium silicate filler content and the nano-hybrid composite material (Grandio SO) with the help of 10-MDP. 

Some studies have reported a linear relationship between the bond strength of the material and the modulus of elasticity [52,53]. With respect to the nanoceramic hybrid technology, GB presents a flexural strength of 250–290 MPa and an elastic modulus of 15.5 GPa. LU has a flexural strength of 200 MPa and a high fracture resistance. HC has a flexural strength of up to 190 MPa and an elastic modulus of 7.8 GPa. The elastic modulus of HC is two times lower than that of the natural dentin (18.6 GPa). Both high and low elastic modulus can lead to incompatibility and premature failure between restorative materials [32,54]. Van Noort et al [55] proved that the higher the elastic modulus, the higher the stresses formed at the edge of the bonding-interface. The Grandio SO material used as a repair composite in this study has a modulus of elasticity of 16.65 GPa. This, in particular, increases the stress formation in the connection interface of HC, which has a low elastic modulus; it may have created lower µTBS values in the HC^G^ and HC^APA^ groups compared to the other groups. 

There is no threshold about the acceptable repair bond strength value. Regarding clinically ideal bonding value, the bonding value of at least 20 MPa has been reported, depending on the composite resin used and the repair method [29,56]. Elsaka [22] found that bond strength values ranging from 15 to 25 MPa are acceptable. In this study, SBU application alone provided acceptable values for the LU^C^ and HC^C^ groups. Similarly, Arpa et al. [18] found the µTBS (54 MPa) values obtained by applying only SBU to the LU material were above the clinically acceptable values. However, this study also showed that proper surface treatment of the restoration material has positive effects when performing a repair procedure.

The shear test, a common way to understand bond strength, often results in cohesive fractures, which limit an accurate assessment and hinder knowledge of bond strength. During the shear test, the specimens are exposed to an uneven distribution of stress. This results in stress concentration in certain areas. In contrast, μTBS is performed with smaller sized specimens and provides a more homogeneous and uniform stress distribution [56]. Accordingly, in this study, μTBS test was conducted. Adhesive failures were detected as the predominant failure pattern, and the rest were mostly cohesive fractures in the direct resin composite. Adhesive-type failure is generally considered to be the best nominal strength-based representation of bond strength. Cohesive failure can be considered a measure of bulk material strength rather than a measure of adhesive bond strength. This is because a complex three-dimensional stress distribution occurring in the bi-material joint of the post-force adhesive bond is difficult to interpret in terms of the quality of bond strength. The mixed type of failure is the failure that encompasses the boundaries of both substrates, but primarily occurs at the adhesive interface [57]. It can be considered as a measure of adhesive bond strength [58]. The adhesive-type failure is dominant in the failure mode analysis, and this indicates that the stress on the specimens is concentrated at the composite resin-substrate interface and the bond strength is obtained as desired [59]. This can be attributed to the use of μTBS as a sensitive test method and the use of thermally-aged, bar-shaped specimens. Bar-shaped specimens and μTBS are more suitable for obtaining the effects of thermal changes on materials and bond strengths [13].

If an intraoral repair of a restoration of unknown material is desired, the current results suggest that the LI and APA+LI method followed by the application of a universal adhesive is recommended for successful repair by clinicians. Since there was no significant difference between all LI and APA+LI groups, the use of a universal adhesive after the application of LI in order to shorten the time spent at the patient’s chairside would provide sufficient bond strength.

This study has a number of limitations. First, only one type of bonding agent was applied to all specimens following surface treatments. Different agents may present different results. Second, the aging cycle was applied only before the repair process. However, how the repair bond strength values would be affected after thermal aging is a conflict of issue. Therefore, further studies are needed.

## 5. Conclusions

Within the limitations of the study, the following conclusions can be reached:

1. Except for the Grandio Blocs control group (18.01 MPa), all surface treatments showed clinically acceptable (≥20 MPa) bond-strength values.
2. Laser irradiation can be applied as an alternative route to bur grinding and airborne particle abrasion recommended for surface roughening in intraoral resin matrix ceramic repair.
3. Considering the micro-tensile bond strength values obtained, it is recommended that airborne particle abrasion + laser irradiation and Single Bond Universal be used as surface treatments for the Grandio Blocs to be repaired, Single Bond Universal without any additional surface treatment for Shofu Block HC, and bur grinding and Single Bond Universal for Lava Ultimate.

## Figures and Tables

**Table 1:** All materials used in the present study [6,7,8,9,10].

Material	Manufacturer	Specification	Composition	Lot Number
Single Bond Universal	3M ESPE, St. Paul, MN, USA	Universal Adhesive	HEMA, 10-MDP, dimethacrylate resins, Vitrebond copolymer, silane, filler, ethanol, water, initiators. pH: 2.7	01026A
Grandio SO	VOCO GmbH, Cuxhaven, Germany	Universal Nano-hybrid Composite	89% *w*/*w* high filler content. 60% functionalized nanoparticles (20–40 nm) in the resin content and glass ceramic.	2109672
Grandio Blocs (GB)	VOCO GmbH, Cuxhaven, Germany	Nano-hybrid Composite	86% *w*/*w* nano silica and barium glass fillers in a polymer matrix. 14% UDMA, DMA.	2014063
Shofu Block HC (HC)	Shofu Dental GmbH, Ratingen, Germany	Resin Nanoceramic	Silica powder, micro fumed silica, zirconium silicate fillers 61% by weight. UDMA, TEGDMA.	071601
Lava Ultimate (LU)	3M ESPE, St. Paul, MN, USA	Resin Nanoceramic	80% silica and zirconia nanoparticles and nanoclusters as filler content. 20% BisGMA, UDMA, BisEMA, TEGDMA.	NA57353

HEMA-2-hydroxyethyl methacrylate; 10-MDP-10-methacryloyloxydecyl dihydrogen phosphate; UDMA-urethane dimethacrylate; DMA-dodecyl dimethacrylate; TEGDMA-triethylene glycol dimethacrylate; BisGMA-bisphenol A diglycidyl ether dimethacrylate; BisEMA-ethoxylated bis phenol A dimethacrylate.

**Table 2:** Two-way ANOVA results.

Source	Type III Sum of Squares	df	Mean Square	F	Sig.
Material-Type (A)	475.920	2	237.960	1.335	0.265
Surface-Treatment (B)	19352.476	4	4838.119	27.147	0.000
A × B	35023.972	8	4377.996	24.565	0.000

**Table 3:** Mean microtensile bond strength (MPa) and ± standard deviation data according to the factors “material” and “surface treatment”.

Material	Surface Treatment	Group	Mean ± Standard Deviation
Grandio Blocs (GB)	Control (C)	GB^C^	18.01 ± 7.88 ^A^
	Bur grinding (G)	GB^G^	63.57 ± 11.22 ^EF^
	Airborne particle abrasion (APA)	GB^APA^	61.88 ± 9.40 ^EF^
	Laser irradiation (LI)	GB^LI^	64.77 ± 5.26 ^EF^
	Airborne particle abrasion + Laser irradiation (APA+LI)	GB^APA+LI^	70.94 ± 6.03 ^F^
Shofu Block HC (HC)	Control (C)	HC^C^	64.35 ± 17.99 ^EF^
	Bur grinding (G)	HC^G^	42.86 ± 17.18 ^BCD^
	Airborne particle abrasion (APA)	HC^APA^	35.98 ± 7.20 ^BC^
	Laser irradiation (LI)	HC^LI^	60.50 ± 12.32 ^EF^
	Airborne particle abrasion + Laser irradiation (APA+LI)	HC^APA+LI^	58.46 ± 16.87 ^DEF^
Lava Ultimate (LU)	Control (C)	LU^C^	28.46 ± 4.92 ^AB^
	Bur grinding (G)	LU^G^	66.09 ± 21.66 ^EF^
	Airborne particle abrasion (APA)	LU^APA^	64.40 ± 21.62 ^EF^
	Laser irradiation (LI)	LU^LI^	49.63 ± 10.54 ^CDE^
	Airborne particle abrasion + Laser irradiation (APA+LI)	LU^APA+LI^	59,66 ± 12.34 ^EF^

Different letters in the same column indicate statistically significant difference (*p* < 0.05).

## Data Availability

Not applicable.
